# Zika Virus: A Systematic Review of Teratogenesis, Congenital Anomalies, and Child Mortality

**DOI:** 10.7759/cureus.34735

**Published:** 2023-02-07

**Authors:** Sara Elena Guerrero Saldivia, Sumedha Unnikrishnan, Yeny Y Chavarria, Adebisi O Akindele, Ana P Jalkh, Aziza K Eastmond, Chaitra Shetty, Syed Muhammad Hannan Ali Rizvi, Joudi Sharaf, Kerry-Ann D Williams, Maha Tariq, Maitri V Acharekar, Prachi Balani

**Affiliations:** 1 Ophthalmology, California Institute of Behavioral Neurosciences & Psychology, Fairfield, USA; 2 Internal Medicine, Government Medical College, Kozhikode, Thrissur, IND; 3 Internal Medicine, Research, California Institute of Behavioral Neurosciences & Psychology, Fairfield, USA; 4 Research, California Institute of Behavioral Neurosciences & Psychology, Fairfield, USA; 5 Family Medicine, Dermatology, California Institute of Behavioral Neurosciences & Psychology, Fairfield, USA; 6 Internal Medicine, California Institute of Behavioral Neurosciences & Psychology, Fairfield, USA; 7 Medicine and Surgery, California Institute of Behavioral Neurosciences & Psychology, Fairfield, USA; 8 Neurology, California Institute of Behavioral Neurosciences & Psychology, Fairfield, USA; 9 Anesthesiology, California Institute of Behavioral Neurosciences & Psychology, Fairfield, USA; 10 Family Medicine, California Institute of Behavioral Neurosciences & Psychology, Fairfield, USA; 11 Gastroenterology, California Institute of Behavioral Neurosciences & Psychology, Fairfield, USA; 12 Internal Medicine, Saint Vincent Hospital, Worcester, USA

**Keywords:** vaccine, herd immunity, mortality, anomalies, zikv

## Abstract

Zika virus infection (ZIKV) was one of the most catastrophic epidemics. ZIKV in nonpregnant women is mild and sometimes asymptomatic. However, infection during pregnancy leads to congenital malformations in the fetus, while maternal signs of infection are preceded by a rash. The maternal-fetal infection begins with a rash that occurs early during pregnancy. The most severe pathologies were related to the first trimester of gestation, including microcephaly, musculoskeletal, genitourinary, craniofacial, ocular, and pulmonary manifestations. The prognosis may not be encouraging. Herd immunity increases CD8^+^ (cytotoxic T-lymphocytes) earlier and decreases in the resolution phase. However, CD4^+^ (T-helper cells) remains higher after infection. Recent ongoing vaccine development shows good immunity, control of the vector (Aedes mosquitoes), and treatment. ZIKV, anomalies, mortality, herd immunity, and vaccine were our main keywords. This systematic review demonstrates the teratogenesis of ZIKV in children, congenital anomalies, mortality, and a view of the future and behavior of ZIKV.

## Introduction and background

Introduction

Zika virus (ZIKV) was initially discovered in Uganda, Zika Forest in 1947 by a rhesus monkey [1]. It is transmitted by the Aedes aegypti mosquitoes [1]. Later, it was discovered by a group of workers and then extended to other humans in Africa and Asia [2]. ZIKV was responsible for several outbreaks on Yap Island of Micronesia in 2007 and the epidemic outbreak in French Polynesia, New Caledonia, the Cook Islands, and Easter Island in 2013 and 2014 [1]. It was not until 2015 that it began spreading to the Americas [3]. In 2015, a massive increase in ZIKV was reported in the Americas, specifically in Brazil [1]. Brazil was the most affected country, with 440,000 to 1.3 million cases [1]. The World Health Organization (WHO) described ZIKV infection as similar to dengue fever and chikungunya by manifesting fever, skin rash, headache, arthralgia, and myalgia [1,2]. The virus can be passed through the placenta with teratogenic effects in the fetus, including central nervous system (CNS) pathologies [4]. Congenital Zika infection (CZS) can be normal or severe; early infection during the first trimester increases the risk for intrauterine growth restriction (IUGR), while late complications have different manifestations [4]. The first and second trimesters have an elevated risk of affecting the development of CNS in the fetus [5]. The external characteristics of CZS are broad, such as microcephaly with cortical atrophy, dysphagia, and epilepsy, which might manifest in early life [4]. Congenital microcephaly is identified by a decrease greater than −2 standard deviation from the mean adjusted for gestational age [6]. The live-born children are not thoroughly studied, but the most severe cases seem related to the first trimester of exposure [4]. For children with CZS, those born a term and at average weight, the risk of death among those with the lowest weight did not differ [4]. Some evidence shows that children might have a death rate of around 10% during the first year of life [6]. It is well known that ZIKV can infect human stem cell-derived neural progenitor cells, resulting in a dysregulation of the cell cycle and leading to an apoptosis cellular and microcephaly [7]. Unfortunately, the limitations on data registration and clinical data were not available. Second, the health service network does not have a protocol for diagnosing ZIKV infections during pregnancy. Therefore, there might be under-reported in the public health system about the fetus who had prenatal exposure to ZIKV. Showing the teratogenic virus infection can be on the fetus might draw attention to primary prevention in women and include maternal screening during pregnancy, herd immunity, and the possibility of vaccines. The purpose of this review attempts the possible future of ZIKV and what we can expect.

Method

**Figure 1:**
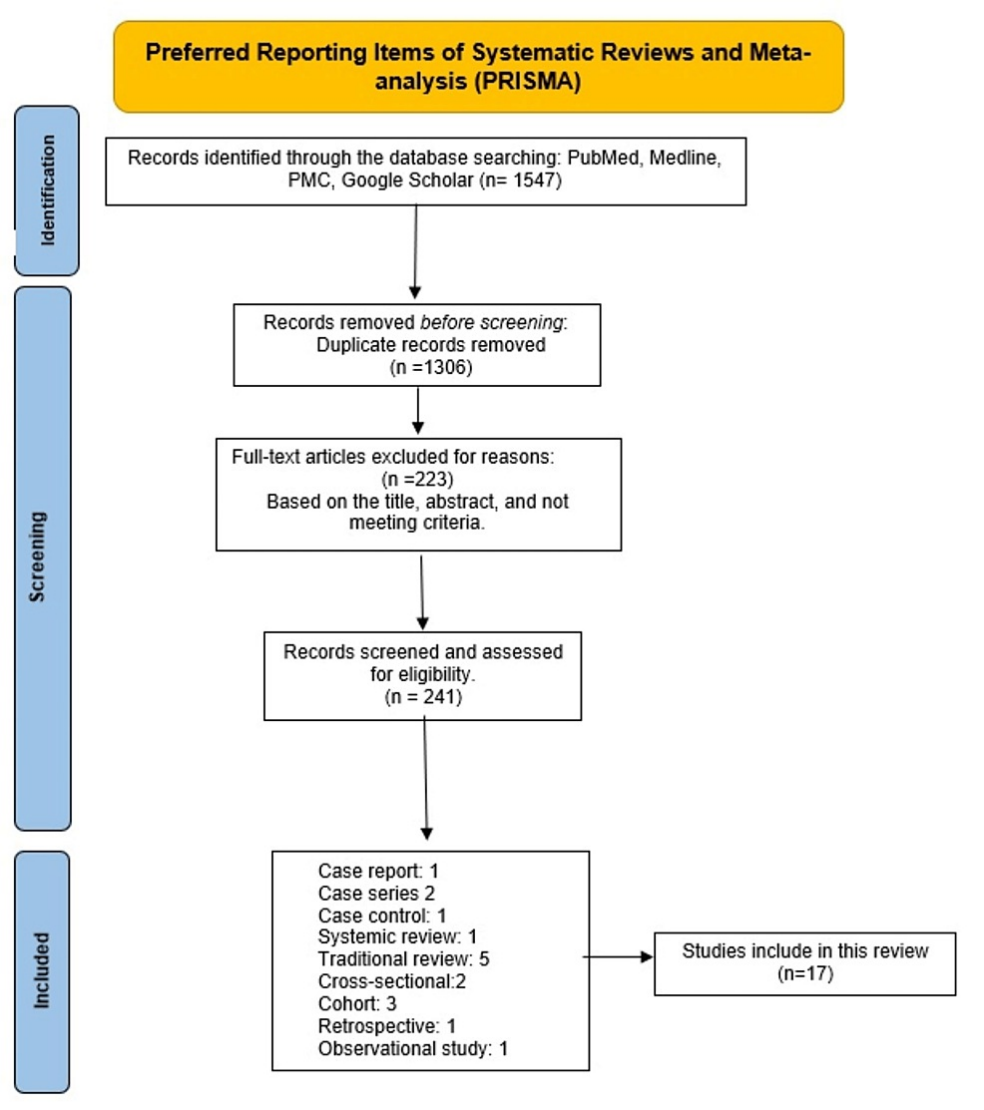
PRISMA: preferred reporting items for systematic reviews and meta-analyses. PMC: PubMed central.

We did this systematic review using preferred reporting items for systematic reviews and meta-analysis (PRISMA 2020) [8]. Figure 1 represents the PRISMA flow diagram delineating the study identification, selection, and inclusion processes used in the present review.

We explored the databases using Medical Subject Heading (MeSH) from PubMed, Medline, Google Scholar, and PubMed central (PCM), and keywords like “anomalies,” “zika virus,” “mortality,” and “vaccine.” We searched the database using these keywords separately and in combination to find relevant studies. We performed a non-automated search on the reference section of included studies and found 1547 articles. Following this, we removed the 1306 duplicates with Microsoft Excel version 10 (Microsoft Corporation, New York, USA). The records were assessed for eligibility. A total of 241 articles were evaluated, and we excluded 223 studies. The following inclusion criteria were implemented for the search: free full text, meta-analysis, clinical trial/observational study, randomized controlled trial, systematic review, English language, PubMed, Medline, Google Scholar, and PCM. The exclusion reasons were obstetric articles, animals instead of humans, socioeconomic articles, opinion-based articles, and no English (French). For the remaining 17 full-text articles, we assessed eligibility. We applied the assessment tools for quality check, namely the Scale for the Assessment of Narrative Review Articles (SANRA) for formal review, the Joanna Briggs Institute (JBI) check tool for case reports, the preferred reporting items for systematic reviews and meta-analyses (PRISMA) for systematic review, and Newcastle for observational studies. We found case reports, systematic reviews, traditional reviews, cross-sectional, observational studies, and cohorts in the studies covering the target areas.

Results

A total of 1547 studies were obtained by scrutinizing the database. The articles were analyzed based on the title and abstract and were filtered, applying the inclusion-exclusion criteria. We removed duplicate studies, assessed 241 records, and applied them to the quality check; we were left with 17 articles. Eleven articles were discussed and reviewed, including 8968 patients with birth defects due to ZIKV. The records reviewed included one retrospective, one case report, two case series, two cross-sectional studies, one case-control study, three cohorts, and one systemic study. They had to meet 70% of the checklist to qualify for the systematic review. 

## Review

Discussion

Zika's Virus Infection During Pregnancy and Its Impact on Neonates

To develop an infection, the virus must go through some essential steps. Firstly, the ZIKV overcomes the local defenses. Then, it infects the cells susceptible to producing more virions and infects the remaining cells and the fetal tissue [7].

One possible alternative to Zika-fetal neuropathogenesis suggested by Klase et al. is the infection of the fetal tissue by ZIKV [7]. The condition of fetal tissue might be an additional factor in the transcytosis of the ZIKV, which goes through the placenta and disseminates in the fetus, mainly affecting the brain [7]. Moreover, one of the most important features is the unidirectional mechanism of infection, where maternal antibodies go from the amniotic fluid to the fetus. The maternal IgG antibodies cross the placenta at week 16 of pregnancy. However, in some cases, it was also found that mothers of infants with microcephaly showed positive ZIKV infection even before the 10th week [7]. Alvarado et al. concluded the infection should occur at 17 weeks of gestation for microcephaly to develop. Additionally, the Zika maternal viremia could remain for 9.9 days. However, pregnant women reported signs of ZIKV infection during the first trimester, followed by a spontaneous abortion at 11 weeks. Identification of the virus in the serum remained positive for 21 days after the first signs of ZIKV were noticed. This conveyed the extended viremia state. In addition, the ZIKV was isolated from fetus tissue, amniotic liquid, and the placenta [7]. Mercado et al. analyzed the amniotic fluid of pregnant women with ZIKV signs. Their description and the analysis of the fluid prenatally and during delivery were congruent with the previous study. Furthermore, the results showed that children of women with ZIKV were associated with congenital birth anomalies (CBA) [9].

The teratogenicity and neuropathology associated with ZIKV might affect fetal growth across the placenta, selective to the central neural system [7]. Consequently, Gallo et al. noticed that most severe brain anomalies, likely microcephaly, were linked with the rash during the first trimester of pregnancy [10]. França et al. said the rash occurred earlier during pregnancy and the head size was smaller at birth [11]. Moreover, they found the last trimester rash in pregnant women was related to brain anomalies despite an average head circumference [11]. Also, Alvarado et al. mentioned the most severe brain pathologies were related to the first trimester of gestation, including microcephaly, hydrocephalus, almost complete agyria, holoprosencephaly, and multiple calcifications in the cortex and subcortical white matter. In addition, they described that the CZS includes musculoskeletal, genitourinary, craniofacial, ocular, and pulmonary manifestations [12]. Comparing the results of de Araujo et al., they obtained microcephaly in all the cases were polymerase chain reaction (PCR) positive for ZIKV, lower weight at birth, and one or more brain anomalies, namely calcification, ventriculomegaly, and lissencephaly [13]. Massetti et al. described the first and second trimesters of gestation with ZIKV infection were related to smaller head size at birth and the followed assessment as more minor for their gestational age, lower weight, motor, and visual anomalies [5]. Similarly, Kikuti et al. obtained congenital cerebral anomalies such as intracranial calcification and ventriculomegaly [14]. However, they claimed that they did not find any statistical significance if the pregnant women had a rash during the first, second, or third trimester of gestation [14].

A list of all the studies included in the review is shown in Table 1.

**Table 1:** Important details of the studies included in this systematic review. ZIKV: Zika virus, CZS: congenital Zika syndrome, PCR: polymerase chain reaction, CBA: congenital birth anomalies, SD: standard deviation.

Study	Author	Year	Type of study	Patient	Purpose of the study	Results	Conclusion
1	Mercado et al. [9]	2020	Retrospective cohort	One hundred thirty-six of amniotic sample	Describe ZIKV test results from amniotic specimens and birth defects.	Sixty-eight women had a ZIKV infection. Fifty-nine women got a ZIKV through testing amniotic fluid. Zika birth defects were found among the children of women with positive viremia.	ZIKV infection can be found in amniotic fluid.
2	Gallo et al. [10]	2020	Systematic review and meta-analysis	509	Analyzed maternal and fetal factors related to microcephaly in children from mothers with ZIKV.	Five hundred and nine children were diagnosed with ZIKV and microcephaly. It is related to infections in the first trimester.	Showed projection of microcephaly due to ZIKV.
3	Massetti et al. [5]	2020	Cross-sectional	65	Describe birth defects in the first and second trimesters.	Twenty women had ZIKV infection during the first trimester, and 11 women had it in the second trimester. Thirty-one children presented with microcephaly.	All the children with microcephaly due to CZS showed poor motor development and visual outcomes.
4	Alvarado et al. [12]	2017	Traditional review	_	Effect of maternal ZIKV on the fetus.	Microcephaly, hydrocephalus, agyria, and multiple calcifications in the cortex were noticed in some fetuses. An autopsy of the brain revealed calcifications, gliosis, apoptosis, and holoprosencephaly. Four fetuses were PCR ZIKV positive. The first trimester is the risker. The development of microcephaly occurs at 17 weeks of gestation.	The brain is the most severely affected.
5	França et al. [11]	2016	Cases-series	1501	Characterized findings such as survival and length in children with CZS.	About 77% of women had a rash in the first trimester, 18% in the second trimester, and 5% in the last trimester. The survival average was of eight days. The average head size was located under SD 0.1.	They found that it reduces the size of the head. The last trimester's rash was related to brain anomalies.
6	Klase et al. [7]	2016	Traditional review	_	Featured the most critical aspects of flavivirus replication and mechanism.	ZIKV passes through the placenta unidirectionally. Maternal IgG goes through the placenta at week 16th. Suggested an incomplete formation of the blood-brain barrier. Growth retardation is observed in the last trimesters.	ZIKV reveals vertical infection patterns and the teratogenic effect during the first trimester.
7	Mlakar et al. [1]	2016	Case report	1	Describe the vertical transmission of ZIKV during pregnancy in the first trimester.	Body weight was in the fifth percentile, and head size was in the one percentile. Placenta weight was reduced. Macroscopic examination found microcephaly, hydrocephalus, agyria, and calcification in the placenta. PCR is positive for ZIKV.	Showed a reduction in head size and decreased placental and body weight.
8	Kikuti et al. [14]	2016	Cases-series	166	Describe the features of children with CBA.	Intracranial calcification is 86.1% of children and 66.9% of children with ventriculomegaly.	Neurological outcome of ZIKV epidemic.
9	de Araujo et al. [13]	2016	Cases-control	Cases 32, control 62	Explanations of microcephaly related to congenital ZIKV.	Eleven cases had a small head and were born with low weight. Twenty-seven patients were investigated by brain imaging, and 11 had one or more anomalies: calcification, ventriculomegaly, and lissencephaly. Thirty-two cases were PCR-ZIKV positive.	The microcephaly is due to CZS.

Factors Affecting Mortality of Children-Prognosis

According to Paxiao et al., the risk of death among children with microcephaly due to ZIKV infection will not differ from those who are born small [4]. However, the total mortality rate went up to 36 months of age in children with CZS, which was 11.3 times higher than in kids without the syndrome [4]. In addition, those kids born after 32 weeks of gestation with CZS were more likely to die, and the most elevated mortality rate was observed in children born a term with ZIKV [4]. Children with CZS were small and five times higher to die, and kids born of normal size for their age had ZIKV infection getting nine times the rate of dying compared to those without [4]. Compared to Costa et al., the highest mortality rate for children with ZIKV was 0-2 years old [6]. They mentioned the rate of death was elevated in the early neonatal period and those who died immediately at born for different causes such as respiratory distress, septicemia, and acute respiratory failure [6]. Also, they found out the mortality was located between lower birth weight (<1500 g) and those who were born <31 weeks of gestation [6].

A list of all the studies included in the review is shown in Table 2.

**Table 2:** Summarizes the causes of death among children infected with intrauterine Zika virus infection. CZS: congenital Zika syndrome.

Study	Author	Year	Type of study	Patient	Purpose of the study	Results	Conclusion
1	Paixao et al. [4]	2022	Retrospective cohort	398	Estimating the mortality of children with CZS and without the syndrome.	The mortality rate is 11.3 times higher. The death rate of those who had CZS and those without was 82.6%. Children born after 32 weeks of gestation with CZS had 14.3 times the chance of dying.	The risk of death among children with CZS will not differ from the smallest.
2	Costa et al. [6]	2020	Retrospective	6059	Investigates the causes of fatality.	Age of death 0-2 years was 92.8%. Under one year was 52.6%. Neonatal period (early neonatal 38.2% and late 14.4%). One year was 7.6%. Immediate causes of death: newborn respiratory failure 12.6%, septicemia 11.9%, and acute respiratory failure 6.9%. Birth weight <1500 g was 16.9%, 1500-2499 48.5%, premature <31 weeks was 10%, 32-36 weeks was 29.9%, and 34-36 weeks was 59.5%.	The death rate was higher among those with lower weight, Apgar <7, and prematurity.

Vaccines and Herd Immunity

ZIKV can be self-limiting in adults, but in newborns, it can lead to a wide of malformations of the CZS. Some mothers and babies develop long T-cell immunity [15].

There are several challenges regarding the future of ZIKV, vaccination, and herd immunity. According to Badolato-Corrêa et al., pregnant women with ZIKV and children exposed to the virus showed long-term immunity related to T-helper (CD4T) cells [15]. However, it was not the same with cytotoxic T-lymphocytes (CD8T) cells for ZIKV, which were increased at the beginning of the disease but eventually went down in the resolution phase [15]. Parker et al. mentioned that cytomegalovirus (HCMV) and ZIKV have different approaches to affecting the mother and the baby [16]. They concluded that the interaction with CD4^+^ cells could be more robust than with CD8^+^ cells. However, the interaction with CD8^+^ cells was more into structural proteins such as Cap and Env [16]. The Flavivirus family, in which ZIKV is part, has an immune process called antibody-dependent enhancement (ADE) [17]. Pierson et al. claimed this process neutralized the ZIKV antibodies with the increase of CD4 and CD8, following the idea of a deoxyribonucleic acid (DNA) vaccine [17]. In addition, Mwaliko et al. specified several experimental vaccines such as DNA, messenger ribonucleic acid (mRNA), peptide-protein, vital-like particles, inactivated-virus, and live-attenuated viruses [18]. Nevertheless, they found the RNA of ZIKV can change the virus proteins quickly, leading to drug resistance [18]. Therefore, additional research is needed.

A list of all the studies included in the review is shown in Table 3.

**Table 3:** Important details of the studies included in this systematic review. ZIKV: Zika virus, ADE: antibody-dependent enhancement, RNA: ribonucleic acid, DNA: deoxyribonucleic acid, CD4T: T-helper cells, CD8T: cytotoxic T-lymphocytes, mRNA: messenger ribonucleic acid, NS1: nonstructural protein 1, NS3: nonstructural protein 3, NS5: nonstructural protein 5, Env: envelope, Cap: catabolite activator protein.

Study	Author	Year	Type of study	Patient	Purpose of the study	Results	Conclusion
1	Badolato-Corrêa et al. [15]	2021	Cross-sectional	21 mothers, 18 children	ZIKV memory T cells during pregnancy.	ZIKV infection showed long-term CD4T protection in mothers and kids. But it was not observed that CD8T cells were against ZIKV in mothers and kids.	Most CD8T cells were found at the beginning of the infection due to the pro-inflammatory effect.
2	Mwaliko et al. [18]	2021	Traditional review	_	Provides a summary of ZIKV development and vaccine current situation.	The DNA, mRNA, peptide-protein, vital vectors, virus-like particles, inactivated-virus, and live-attenuated virus vaccines develop a humoral and cellular and decreased viremia and RNA in vitro and in vivo.	RNA ZIKV seems to lead to rapid mutations in the structural proteins of the virus. At present, there are no vaccines or drugs approved for humans.
3	Parker et al. [16]	2020	Traditional review	_	Maternal and fetal T cells, natural killer (NK), and the interaction with ZIKV and cytomegalovirus (HCMV)	Showed responses from CD4^+^ and CD8^+^ cells. It could be seen stronger response with CD4^+^ cells to structural proteins NS1, NS3, and NS5, and CD8^+^ cells were more to Cap and Env proteins and the importance of interferon (INF), which influences ZIKV replication.	The outcome of viral interaction and replication in maternal-fetal cells.
4	Pierson et al. [17]	2016	Traditional review	_	Investigate the rapid advance in Zika immunity and vaccine development.	ZIKV immunity is an ADE process. This process has been shown in vitro and neutralized flavivirus antibodies. ZIKV vaccine DNA is more safety. This vaccine is now in phase I of the clinical trial. The passive transference of antibodies with CD4 and CD8 should be protection enough.	Zika's vaccine is in a clinical trial, However, it promises the antibodies' creation.

Limitations of this systematic review

This systematic review was limited in several aspects. Some articles were not available for a long-term following on these babies with ZIKV and their development over a lifetime period. The difficulty to access databases like Scopus and Web Science was paid or needed institutional access, and those papers that must be paid we did not get access as well. Finally, the vaccine is still in a clinical trial without being tested on humans.

## Conclusions

The ZIKV was a pandemic that affected a million pregnant women leading to their babies being born with various anomalies. We found the tropism of ZIKV for fetal neural tissue was the primary affection. However, the teratogenicity associated with ZIKV occurred early during pregnancy, especially in the first and second trimesters. Microencephaly and brain calcification are outcomes of congenital Zika virus infection. Life prognosis is not so encouraging, children do not go over two years old. Moreover, the death rate was related to lower birth weight and those born prematurely. Herd immunity has been associated with the increase of CD4T cells, similarly to the increase in CD8T cells in the early stages of the disease, and then it went down in the resolution phase. The future is promising: the vaccine is now in phase I of the clinical trial and shows that the passive transference of antibodies that have CD4^+^ and CD8^+^ must be adequate for protection.
